# Eruption Chronology in Children: A Cross-sectional Study

**DOI:** 10.5005/jp-journals-10005-1450

**Published:** 2017-02-27

**Authors:** Neha Verma, Arpana Bansal, Parimala Tyagi, Ankur Jain, Utkarsh Tiwari, Ruchika Gupta

**Affiliations:** 1 Postgraduate Student, Department of Pediatric and Preventive Dentistry, People’s Dental Academy, Bhopal, Madhya Pradesh, India; 2 Reader, Department of Pedodontics and Preventive Dentistry, People’s Dental Academy, Bhopal, Madhya Pradesh, India; 3 Professor, Department of Pedodontics and Preventive Dentistry, People’s Dental Academy, Bhopal, Madhya Pradesh, India; 4 Reader, Department of Pedodontics and Preventive Dentistry, People’s Dental Academy, Bhopal, Madhya Pradesh, India; 5 Senior Lecturer, Department of Pedodontics and Preventive Dentistry, People’s Dental Academy, Bhopal, Madhya Pradesh, India; 6 Postgraduate Student, Department of Community and Preventive Dentistry, People’s Dental Academy, Bhopal, Madhya Pradesh, India

**Keywords:** Delayed eruption, Eruption, Tooth.

## Abstract

**Aims and objectives:**

The purpose of this study is to determine the appropriate reference standard for eruption timing of primary teeth in infants and preschool children of Bhopal city and to determine the role of various factors affecting the eruption of primary dentition.

**Materials and methods:**

A cross-sectional study was conducted among the infants and preschool children (4-36 months) attending the local government or private hospitals, and vaccination centers. Prior to the study, Institutional Ethical Committee clearance and informed written consent from the parents were obtained. The data were collected from full-term infants and preschool children of 4 to 36 months from Bhopal city. Oral examination was done under adequate natural light by a single examiner using mouth mirror and probe. Teeth present in the oral cavity were noted by using Federation Dentaire Internationale system of nomenclature in the preformed pro-forma. The teeth were considered as erupted, when any part of its crown had penetrated the gingiva and was visible in the oral cavity. Height, weight, birth weight, and other close-ended questions in questionnaire were asked from parents.

**Results and conclusion:**

The data collected were statistically analyzed and it was observed that significant relation exists between tooth eruption and birth weight, feeding habits, socioeconomic status, and body mass index (BMI). Based on the findings, it may be concluded that Indian children experienced delayed eruption of primary teeth when compared with children of different countries and standard norms.

**How to cite this article:**

Verma N, Bansal A, Tyagi P, Jain A, Tiwari U, Gupta R. Eruption Chronology in Children: A Cross-sectional Study. Int J Clin Pediatr Dent 2017;10(3):278-282.

## INTRODUCTION

Tooth eruption is the process by which developing teeth emerge through the soft tissues of the jaws and the overlying mucosa to enter the oral cavity, contact the teeth of opposing arch, and function in mastication.^1^

The term "eruption" is derived from the Latin word "erupptione," which means output with momentum.^2^ It is a continuous process that ends only with the loss of tooth. Eruption of deciduous teeth, their exfoliation followed by eruption of permanent dentition is an orderly, sequential, and age-specific event and is considered as an important milestone during child’s development.^3^

Evolution of the human race has seen many changes in the living habits, food habits, and oral hygiene habits over a span of thousands of years, which may have influenced the eruption of teeth as well.^4^ Tooth eruption recognized as an aspect of human growth and development could possibly be influenced by number of factors that can be both physiological and pathological like growth, caries, malnutrition, genetics, etc.^5^

Estimation of eruption schedule can be a very valuable asset in diagnosis and treatment planning during developmental years.^6^ Significant deviations from accepted norms of eruption time are often observed in clinical practice. Premature eruption has been noted, but delayed tooth eruption is the most commonly encountered deviation from normal eruption time.

In a developing country like India, a large number of people are illiterate and have no knowledge or records of their date of birth which is required by law-enforcing agencies in matters like criminal responsibilities, identification, consent, employment, etc. Age estimation is also required for admission purposes at the time of schooling, joining services, and during retirement. Hence, scientific determination of age is very important.^7^

Sequential and timely eruption of teeth is critical in overall development of the child. Variations can occur due to various reasons, but eruption delay of more than 2 years should be investigated.

Malnutrition and poor nutrition in early childhood affects tooth eruption and results in the delayed emergence of the teeth.

So a study was undertaken with the aim to determine the teeth eruption chronology and sequence of eruption in primary dentition and to assess the role of various factors in teeth eruption.

## OBJECTIVES

- To determine the teeth eruption chronology and the eruption sequence of primary dentition.
- To determine the status of teeth in relation to birth weight and feeding habits on teeth eruption and their present status.
- To determine the status of teeth in relation to BMI and the effect of socioeconomic status in relation to present teeth status.

## MATERIALS AND METHODS

The study was cross-sectional in nature and was conducted among 4 to 36 months old children selected from the government and private hospitals of Bhopal city, Madhya Pradesh, India. Prior to the survey, permission to conduct the research and ethical clearance was obtained. This study comprises a total of 1,601 subjects from both sexes. The cases were taken from outpatient departments of government and private hospitals in Bhopal city. Only those cases were considered whose records were available with date of birth and birth weight from immunization card.

The data collection was done by a single trained and calibrated investigator to avoid interexaminer variability. A survey pro forma and questionnaire with close-ended questions was designed in order to collect reliable well-defined information from parents. The questionnaire collected information on demographic details, feeding or dietary habits, and socioeconomic status. Other information like birth weight, date of birth were recorded from immunization card.

Statistical analysis was done using Statistical Package for the Social Sciences (version 20; Chicago Inc., USA). Data comparison was done by applying specific statistical tests to find out the statistical significance of the comparisons. Significance level was fixed at p < 0.05.

## RESULTS

Out of 1,601 subjects, 903 were males and 698 were females. It was observed that among all study subjects mandibular central incisor was the first tooth and maxillary second molar was the last tooth to erupt. The sequence of eruption observed was as follows (Table 1):

Mandibular central incisor < maxillary central incisor < maxillary lateral incisor < mandibular lateral incisor< maxillary first molar < mandibular first molar < maxillary canine < mandibular canine < man-dibular second molar < maxillary second molar. This sequence of eruption was same for boys as well as for girls, whereas eruption timing showed significant difference among the gender. However, it was noticed that the average time period required for complete teeth eruption was 22 months both for boys as well as girls.

Table 2 depicts the eruption age of deciduous man-dibular and maxillary teeth among the study subjects.

On comparing the distribution of teeth according to the present teeth status of the children, it was noticed that as the birth weight increases more number of teeth were present. It was found that there was a statistically significant difference between the groups [according to birth weight as per the World Health Organization (WHO)] (p = 0.001; Table 3).

**Table 1:** **Table 1:** Eruption timing of deciduous mandibular and maxillary teeth among study subjects

*Tooth*		*Eruption age in boys (mean ± SD)*		*Eruption age in girls (mean ± SD)*		*Common eruption age (mean ± SD)*		*Statistical inference*	
Mandibular central incisor		336.86 ± 107.23		350.56 ± 97.44		343.11 ± 102.83		t-value = 2.63	
		(11.2 months)		(11.7 months)		(11.4 months)		p-value = 0.008	
Mandibular lateral incisor		457.17 ± 102.89		431.35 ± 106.30		445.29 ± 104.66		t-value = 4.90	
		(15.2 months)		(14.3 months)		(14.8 months)		p-value = 0.0001	
Mandibular canine		694.14 ± 134.86		711.26 ± 129.01		701.46 ± 132.40		t-value = 2.56	
		(23.1 months)		(23.7 months)		(23.3 months)		p-value = 0.0104	
Mandibular first molar		587.06 ± 131.00		549.66 ± 105.36		570.59 ± 121.30		t-value = 6.15	
		(19.5 months)		(18.3 months)		(19.03 months)		p-value = 0.0001	
Mandibular second molar		893.68 ± 130.45		898.39 ± 131.12		895.85 ± 130.57		t-value = 0.71	
		(29.8 months)		(29.9 months)		(29.8 months)		p-value = 0.474	
Maxillary central incisor		384.86 ± 101.91		386.43 ± 84.72		385.67 ± 93.02		t-value = 0.33	
		(12.8 months)		(12.8 months)		(12.8 months)		p-value = 0.74	
Maxillary lateral incisor		432.93 ± 103.79		423.29 ± 92.32		428.96 ± 99.130		t-value = 1.93	
		(14.4 months)		(14.1 months)		(14.3 months)		p-value = 0.05	
Maxillary canine		681.96 ± 132.53		692.06 ± 137.60		686.41 ± 134.64		t-value = 1.48	
		(22.7 months)		(23.06 months)		(22.8 months)		p-value = 0.13	
Maxillary first molar		571.03 ± 123.62		540.95 ± 107.06		558.62 ± 117.66		t-value = 5.11	
		(19.03 months)		(18.03 months)		(18.6 months)		p-value = 0.0001	
Maxillary second molar		894.89 ± 122.69		913.76 ± 129.79		902.82 ± 125.85		t-value = 2.97	
		(29.8 months)		(30.4 months)		(30.1 months)		p-value = 0.00	

SD: Standard deviation

**Table 2:** **Table 2:** Mean age of teeth eruption in overall study subjects (in months)

*Tooth*		*Maxillary (boys)*		*Mandibular (boys)*		*Maxillary (girls)*		*Mandibular (girls)*		*Maxillary*		*Mandibular*	
Central incisor		8-13		7-11		8-13		8-11		8-13		8-12	
Lateral incisor		13-14		14-15		13-14		14-15		13-14		13-14	
Canines		20-23		20-23		19-23		19-23		18-24		19-24	
First molar		16-19		17-20		15-19		16-19		15-18		15-18	
Second molar		24-30		24-30		23-30		24-30		24-30		24-30	

**Table 3:** **Table 3:** Distribution of teeth according to the birth weight of the children

				*Teeth status*									
*Variable*		*Groups (as per WHO)*		*Absent*		*Present*		*Total cases*		X^2^		*p-value*	
		Severe underweight		214 (66.4%)		108 (33.5%)		322					
		Underweight		172 (64.1%)		96 (35.8%)		268					
Birth weight		Normal		414 (59%)		287 (40.9%)		701		16.88		0.001	
		Overweight		160 (51.6%)		150 (48.3%)		310					
		Total		960		641		1601					

**Table 4:** **Table 4:** Distribution of status of teeth according to feeding habits

				*Teeth status*									
*Variable*		*Type of feeding habits*		*Absent*		*Present*		*Total*		X^2^		*p-value*	
		Breastfeeding		724 (58.9%)		504 (41%)		1228					
Feeding habits		Bottle feeding		57 (67%)		28 (32.9%)		85		2.875		0.238	
		Breast and bottle feeding both		179 (62.1%)		109 (37.8%)		288					
		Total		960		641		1601					

**Table 5:** **Table 5:** Distribution of teeth according to BMI

				*Teeth status*									
*Variable*		*Groups (as per WHO)*		*Absent*		*Present*		*Total*		X^2^		*p-value*	
		Severe underweight		179 (71%)		73 (28.9%)		252					
BMI		Underweight		181 (56.9%)		137 (43%)		318					
		Normal		452 (57.6%)		332 (42.3%)		784		15.8		0.001	
		Overweight		148 (59.9%)		99 (40%)		247					

It was observed that when feeding habits were compared with age-appropriate teeth present, more number of teeth were present in those children who were only breastfed (41%) and least number of teeth were seen in only bottle-fed group (32.9%). Those children who received both breastfeeding and bottle feeding had more number of teeth (37.8%) than those who received only bottle feeding (32.9%). It was also noticed that they had less number of teeth than the group of children who were breastfed alone (41%). However, the result was not statistically significant (p = 0.238; Table 4).

When BMI was compared with age-appropriate teeth present in children, it was observed that the severe underweight group children had least number of age-appropriate teeth (28.9%) present compared with normal (42.3%), underweight (43%), and overweight group (40%), whereas on comparing the overweight children with other groups, it was observed that, though, they had more number of age-appropriate teeth (40%) than the severe underweight children (28.9%). It was also noted that they had less number of teeth than the normal and underweight group. However, the result obtained was statistically significant (p = 0.001; Table 5).

When socioeconomic status was compared with present teeth status of children, it was observed in our study subjects that as status increased more number of age-appropriate teeth were present and vice versa. In upper socioeconomic group, it was observed that 50.9% of age-appropriate teeth were present, which was highest among all classes. The result obtained was statistically significant in the present strata (p = 0.001; Table 6).

## DISCUSSION

Eruption of deciduous teeth, their exfoliation followed by the eruption of succedaneous permanent dentition is an orderly, sequential, and age-specific event. Variation in tooth eruption is found to be multifactorial.^2^

**Table 6:** **Table 6:** Distribution of teeth according to socioeconomic status

				*Teeth status*									
*Variable*		*Class*		*Absent*		*Present*		*Total*		x^2^		*p-value*	
		Lower		1 (100%)		0 (0%)		1					
		Upper lower		115 (73.7%)		41 (26.2%)		156					
Socioeconomic status		Middle		228 (62.2%)		138 (37.7%)		366		18.8		0.001	
		Upper middle		591 (57.5%)		436 (42.4%)		1027					
		Upper		25 (49%)		26 (50.9%)		51					

Since large variability is observed in previous studies, it is preferable not to adopt references from other countries as our standard as Indians differ from them racially, culturally, and environmentally.

In the present study, average age of eruption of deciduous central incisor was 11.4 ± 3.43, whereas in the study conducted by Singh et al^8^ in Amritsar, India, the average age of eruption of central incisors was 8.67 months, which showed delayed eruption in our population. The deciduous dentition completely erupted by 30 months, which was in congruent with our study.

When maxillary and mandibular arch were compared, there was a tendency for lateral incisor, canines, and first molar to be chronologically advanced in maxilla when compared with mandibular counterparts, while central incisors and second molars emerged earlier in mandible. Results are in accordance with Rao et al^9^ in relation to maxillary central, lateral incisors, first, and second molars.

Further, our finding of earlier eruption of second molars in mandible for both boys and girls and tendency of canines and first molars to be chronologically advanced in the maxilla is in agreement with many authors.^3,6,7^

In our study initially, boys showed early eruption of teeth than girls but deciduous dentition was completed in both of them at the same time. It was observed that there was no significant difference between the eruption timing in boys and girls for both maxilla and mandible. Therefore, the combined average for the entire sample was considered. Similar results were obtained by Holmen and Yamaguchi^10^ and Gupta et al^6^ who also observed no difference in the eruption timing between girls and boys.

Sequence of eruption was same in our study, as observed in previous studies, though the timing of eruption was delayed. A catchup in time of teeth eruption was seen in girls during eruption of lateral incisors and first molars.

It was seen that dental eruption and skeletal growth are strongly associated with each other.^11^ Eruption of teeth is found to be positively related to somatic growth. Very few studies have related the eruption timing with birth weight, BMI, feeding habits, and socioeconomic status. Malnutrition and poor nutrition in early childhood affects tooth eruption and results in delayed emergence of teeth.

So keeping this in mind here we have considered factors like birth weight, BMI, and socioeconomic status and observed the effect of these factors on teeth eruption. We have also seen the effect of feeding habits on the number of teeth erupted in children.

Birth weight has been assumed to be related to the rate of dental development. It was observed that low-weight children have dental delay according to lower weight, length, and head circumference at birth and may also present delayed motor skills.^12^

Considering the chronological age, in our study very low birth weight infants (below 2 kg) showed a significant delay in the eruption of the deciduous tooth when compared with low birth weight infants and normal birth weight infants. Normal (2.4-3.3 kg) and overweight children (>3.3 kg) at birth showed greater percentage of age-appropriate teeth. It can be concluded that birth weight has an influence on teeth eruption and has reverse linear relation, i.e., if birth weight was less, the teeth eruption timing was increased and if birth weight was more, eruption timing was less.

The results of our study are in accordance with some studies^13^ and in contrast with Shuper et al^14^ who did not establish any significant relation with birth weight and deciduous teeth eruption. Andrade and Bezerra^15^ did not find any delay in eruption of deciduous teeth.

When teeth eruption was compared with the feeding habits during the first year of life, it was observed that the more number of age-appropriate teeth were present in children who were breastfed, than those who were both breast as well as bottle fed and least number of age-appropriate teeth were seen in only bottle feeding group but the result was not statistically significant.

On comparing feeding pattern with teeth eruption, similar results were observed by Holman and Yamagu-chi^10^ and Aziz^16^ who indicated that delayed emergence of teeth was seen in children who were not breastfeeding. Whereas in contradiction, Folayan et al^17^ failed to establish any link between eruption timing and duration of breastfeeding.

The reason may be attributed to breastfeeding that it contributes an important influence on thrust and growth of mandible^18^ and also to nutritional advantages of the breastfeeding because the milk of the mother has minerals, such as calcium and phosphorous and good vitamins, especially vitamin A and D that is very important in teeth development and formation. It was also observed that children who are breastfed receive milk of a controlled composition, which is beneficial for dental health.

In the present study, a significant positive correlation was seen between BMI and age-appropriate teeth present in children. It was observed that malnourished children, i.e., those who had lower BMI (severe underweight) has lesser number of teeth present as compared with children having normal weight or underweight. This shows that BMI or present weight of child has direct relationship with the teeth eruption.

The results of our study are in accordance with other studies done on permanent teeth, which observed that eruption time increases with decrease in BMI value.^19^

Body mass index can also be related to socioeconomic status, as in our study it is observed that as socioeconomic status increases, more number of age-appropriate teeth is present. Poor children are more frequently affected by malnutrition and will show delayed emergence of teeth.

## CONCLUSION

Indian children experienced delayed eruption of primary teeth. The present study provides the baseline data about the eruption chronology and associated factors in the primary teeth eruption. Furthermore, it is recommended that longitudinal studies are required on a larger scale, to assess the role of various others factors affecting dental eruption chronology in primary dentition.

## References

[1] Avery JK., Steele PF., Avery N. (2002). Oral development and histology..

[2] Neto PG, Falcao MC (2014). Eruption chronology of the first deciduous teeth in children born prematurely with birth weight less than 1500g. Rev Paul Pediatr.

[3] Peedikayil FC (2011). Delayed tooth eruption. e-J Dent.

[4] Lakshmappa A, Guledgud MV, Patil K (2011). Eruption times and patterns of permanent teeth in school children of India. IJDR.

[5] Soliman NL, El-Zainy, Hassan RM, Aly RM (2012). Relationship of deciduous teeth emergence with physical growth. IJDR.

[6] Gupta A, Hiremath SS, Singh SK, Poudyal S, Niraula SR, Baral DD, Singh RK (2007). Emergence of primary teeth in children of Sunsari District of Eastern Nepal. MJM.

[7] Aggarwal KK, Kaur A, Kumar R (2011). Chronological pattern of eruption of permanent teeth in the adolescent age group in Patiala district (Punjab). J Punjab Acad Forensic Med Toxicol.

[8] Singh N, Sharma S, Sikri V, Singh P (2000). To study the average age of eruption of primary dentition in Amritsar and surrounding area. J Indian Dent Assoc.

[9] Rao A, Rao A, Shenoy R, Ghimire N (2014). Changing trends in tooth eruption-survey among children of Mangalore, India. IJAR.

[10] Holman DJ, Yamaguchi K (2005). Longitudinal analysis of deciduous tooth emergence: IV. Covariate effects in Japanese children. Am J Phys Anthropol.

[11] Hussin AS, Mokhtar N, Naing L, Taylor JA, Mahmood Z (2007). The timing and sequence of emergence of permanent teeth in Malay school children in Kota Bharu, Malaysia. Arch Orofac Sci.

[12] Fadavi S, Punwani IC, Adeni S, Vidyasagar D (1992). Eruption pattern in the primary dentition of premature low-birth-weight children. ASDC J Dent Child.

[13] Seow WK, Humphrys C, Mahanonda R, Tudehope DI (1988). Dental eruption in low birth-weight prematurely born children, a controlled study. Pediatr Dent.

[14] Shuper A, Sarnat H, Mimouni F, Mimouni M, Varsano I (1985). Deciduous tooth eruption in Israeli children. A cross-sectional study. Clin Pediatr (Phila).

[15] Andrade IR, Bezerra AC (1998). Estudo longitudinal comparativo da cronologia de erupcjaoemcriancjas. J Bras Odonto Pediatr Odontol Bebe.

[16] Aziz HK (2010). Age estimation of first deciduous tooth and sequence of eruption for the primary dentition in relation to the nursing habits among the kerbala children. J Kerala Univ.

[17] Folayan MO, Oziegbe EO, Esan AO (2010). Breastfeeding, timing and number of erupted teeth in first twelve months of life in Nigerian children. Eur Arch Paediatr Dent.

[18] Westover KM, DiLoreto MK, Shearer TR (1989). The relationship of breastfeeding to oral development and dental concerns. ASDC J Dent Child.

[19] Alvarez JO, Navia JM (1989). Nutritional status, tooth eruption and dental caries: a review. Am J Clin Nutr.

